# Thoracoscopic Plication for Bilateral Eventration of Diaphragm in a Neonate

**Published:** 2015-07-01

**Authors:** Saravanan N, Ramalingam R, Subramanian H

**Affiliations:** Sri Gokulam Hospitals, Salem, India

**Dear Sir**

A 6-day-old female baby delivered by Caeserean section was admitted for respiratory distress. On admission, the baby was tachypnoeic, respiratory rate more than 60/ min with cyanosis requiring oxygen of 8 l/min to maintain satisfactory O2 saturation. An X-ray chest and abdomen taken, was suggestive of bilateral eventration of diaphragm (Fig. 1). An USG and CT scan confirmed the diagnosis. The ECHO done was normal.

**Figure:**
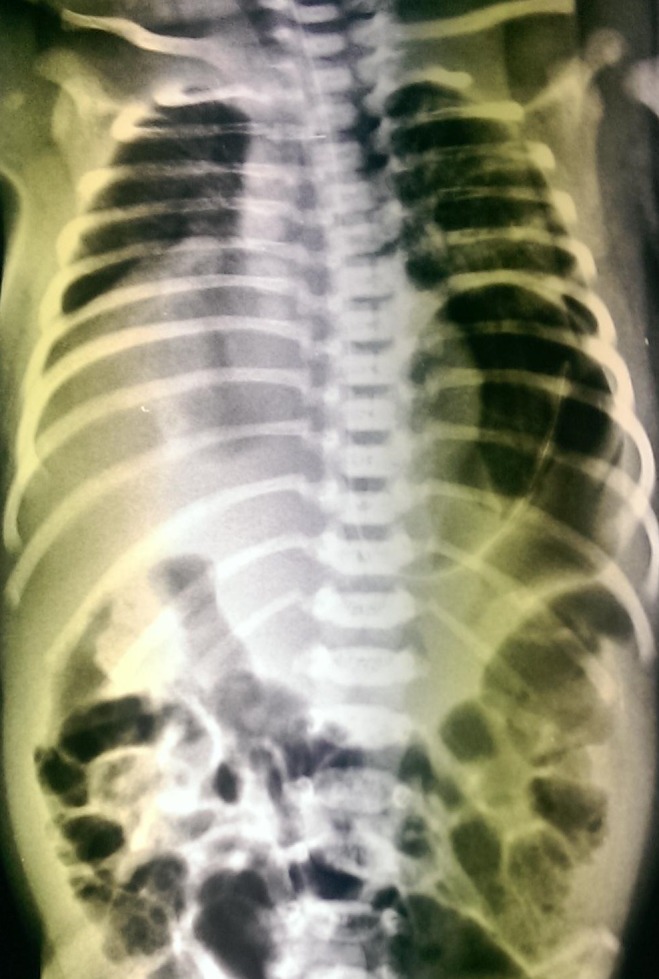
Figure 1: Bilateral eventration of diaphragm.

Baby was taken up for thoracoscopic corrective procedure. It was decided randomly to operate on the left side first. With the baby in lateral decubitus position, a 5mm optical port was introduced for 30 0 telescope in the mid -axillary line at 4th inter costal space. Two additional working ports of 5 mm were introduced in the anterior and posterior axillary lines at 5th inter costal space. Thoracoscopy was done with CO2 at a flow rate of 0.50-1 l/min with pressure 5-6 mm Hg. The eventration was picked up with forceps, carefully avoiding underlying structures. Plication was done with 2-0 polypropylene interrupted sutures tied extracorporeally and pushed down with knot pusher. No clamp or retractor was used. An intercostal drainage tube was introduced, and the baby recovered well. No ventilatory support was needed. Post-operative X-ray showed left hemi diaphragm at normal level. But on right side it had gone further up, may be due to increased intra- abdominal contents. The baby was recovering well, started on tube feeds, but still needed 1-2 lit/min of oxygen. So it was decided to go for right side repair on 6th postoperative day. Thoracoscopic plication of right side diaphragm was done in the same way. Baby had an uneventful recovery. Both ICDs were removed and baby was discharged after 5 days. Baby is on follow up for more than 6 months and is doing well.

Eventration diaphragm is a disorder in which all or part of diaphragmatic muscle is replaced by fibro-elastic tissue [1,2]. It can be congenital or acquired. Bilateral congenital eventration of diaphragm is a relatively rare occurrence [1]. Minimally invasive procedures are being used for the treatment of eventration for the last 2 decades [2,3]; the advantages quoted in favour of thoracoscopic over open surgical approach clinical reduction in perception of pain, less post-operative morbidity and dysfunction[4] To the best of our knowledge, only few references could be gathered about bilateral eventration and none describing thoracoscopic management for bilateral eventration in a new born.[1,5]

## Footnotes

**Source of Support:** Nil

**Conflict of Interest:** None

